# Acceptability of a Health Care App With 3 User Interfaces for Older Adults and Their Caregivers: Design and Evaluation Study

**DOI:** 10.2196/42145

**Published:** 2023-03-08

**Authors:** Joo Chan Kim, Saguna Saguna, Christer Åhlund

**Affiliations:** 1 Division of Computer Science Department of Computer Science, Electrical and Space Engineering Luleå University of Technology Skellefteå Sweden

**Keywords:** Internet of Things, health monitoring, older adults, augmented reality, user experience, independent living, design study, mobile phone

## Abstract

**Background:**

The older population needs solutions for independent living and reducing the burden on caregivers while maintaining the quality and dignity of life.

**Objective:**

The aim of this study was to design, develop, and evaluate an older adult health care app that supports trained caregivers (ie, formal caregivers) and relatives (ie, informal caregivers). We aimed to identify the factors that affect user acceptance of interfaces depending on the user’s role.

**Methods:**

We designed and developed an app with 3 user interfaces that enable remote sensing of an older adult’s daily activities and behaviors. We conducted user evaluations (N=25) with older adults and their formal and informal caregivers to obtain an overall impression of the health care monitoring app in terms of user experience and usability. In our design study, the participants had firsthand experience with our app, followed by a questionnaire and individual interview to express their opinions on the app. Through the interview, we also identified their views on each user interface and interaction modality to identify the relationship between the user’s role and their acceptance of a particular interface. The questionnaire answers were statistically analyzed, and we coded the interview answers based on keywords related to a participant’s experience, for example, ease of use and usefulness.

**Results:**

We obtained overall positive results in the user evaluation of our app regarding key aspects such as *efficiency*, *perspicuity*, *dependability*, *stimulation*, and *novelty*, with an average between 1.74 (SD 1.02) and 2.18 (SD 0.93) on a scale of −3.0 to 3.0. The overall impression of our app was favorable, and we identified that “simple” and “intuitive” were the main factors affecting older adults’ and caregivers’ preference for the user interface and interaction modality. We also identified a positive user acceptance of the use of augmented reality by 91% (10/11) of the older adults to share information with their formal and informal caregivers.

**Conclusions:**

To address the need for a study to evaluate the user experience and user acceptance by older adults as well as both formal and informal caregivers regarding the user interfaces with multimodal interaction in the context of health monitoring, we designed, developed, and conducted user evaluations with the target user groups. Our results through this design study show important implications for designing future health monitoring apps with multiple interaction modalities and intuitive user interfaces in the older adult health care domain.

## Introduction

### Background

According to a United Nations report, the number of people aged ≥65 years in 2020 was approximately 727 million, which is expected to increase to 1.5 billion by 2050 [1]. As the proportion of older adults increases, the demand for older adult care services increases [2,3]. In particular, older adults who live independently require care because of physical and mental health vulnerabilities such as physical constraints, poverty, loneliness, and depression [1]. Their relatives who live independently have difficulty visiting them every day because of distance and time issues. Therefore, older adult care services to improve life satisfaction are necessary. However, the burden on caregivers and relatives keeps growing owing to the aging and increase in the older adult population. According to the American Association of Retired Persons and National Alliance for Caregiving report in 2020 [3], 18% of caregivers covered multiple people in 2015. This ratio increased by 6% over 5 years to 24%. In addition, 54% of caregivers were aged >50 years, and 21% of family caregivers (ie, relatives) reported that caregiving had worsened their health. This phenomenon has worsened because of the pandemic [4,5]. In this context, the necessity of assistive technology to support relatives and caregivers in reducing their burden has continuously grown.

To support caregivers and relatives, the latest status information of an older adult can be provided by an Internet of Things (IoT)–based system. IoT is a technology widely used for collecting data about a person and their environment to enable the system to understand the information of their context.

For example, a sensor attached to a human body could work as a heart rate monitor [6], and in another case, a sensor can read air quality pollutants to work as an air quality sensor [7]. As data need an interface to be delivered to a user, efficient data delivery is as essential as data collection. Augmented reality (AR) draws interest from researchers as a technology that could enhance user engagement [8] and enrich data presentation for better accessibility [9]. The properties of both technologies are attractive; hence, research to improve the merits of IoT and AR has been conducted by combining them since those 2 technologies gained attention [10-12].

There is a need for more research on IoT platform–based AR apps, especially regarding users’ perception of an app’s user interfaces (UIs) and acceptance of the technologies used in the context of health monitoring of an older adult by caregivers and relatives. For example, in the *Internet of Things within health and care* (iVO) project [13], older adults’ activities are sensed by IoT devices, and anomalous events are reported to their relatives via SMS text message [14]. However, efficiently conveying comprehensive information about an older adult’s state to their relatives and caregivers could be done with well-designed UIs rather than SMS text messaging services. In this case, the user experience of the app and user acceptance of the app’s UI with the technologies used should be analyzed based on the user’s role to understand the effectiveness factor. Furthermore, AR is useful for visualizing data. Hadj Sassi and Chaari Fourati [15] showed that displaying real-world data on a 3D AR map identical to the real world is beneficial in understanding the data. However, their user evaluation focused on UIs’ usability related to performing a task and generic user experience. On top of the user experience evaluation, an in-depth analysis of user acceptance regarding interaction modalities and data presentation designs depending on the users’ characteristics (eg, age, gender, and experience) is needed.

Regarding user acceptance, the definition varies based on the purpose of use [16]. Technology acceptance model, developed by Davis [17] is a widely used approach to measure acceptance [16], and it proposes that user acceptance is determined 2 factors: “perceived usefulness” and “perceived ease of use” [17]. “Perceived usefulness” means a user’s perception of the technology, whether it is helpful for their task. “Perceived ease of use” is a user’s feeling of how easy it is to use the technology. These 2 factors influence a user’s belief about the technology, which determines acceptance and use [17]. In addition, a user’s characteristics, such as age, gender, experience, and voluntariness of use, can affect their perception of the technology and, hence, influence user acceptance [18]. In our study, we used the definition of user acceptance by Dillon and Morris [19]: “the demonstrable willingness within a user group to employ information technology for the tasks it is designed to support.” On the basis of this definition, we examined the reason for preference by users in terms of “perceived usefulness” and “perceived ease of use” depending on their characteristics, especially on their role (eg, caregiver, relative, and older adult), from user evaluations to demonstrate a user’s willingness and, thus, the user acceptance regarding interaction modalities and data presentation designs.

### Objectives

Our design study aimed to conduct user evaluations on both the app and its 3 different UIs designed for caregivers, relatives, and older adults to identify the app’s user experience and the factors that affect user acceptance of each UI depending on their characteristics, especially on the participants’ role. In this study, we grouped caregivers and relatives into 1 category, “caregivers,” and separated them based on whether they had training experience in health care services as experts. Accordingly, relatives were labeled as “informal caregivers,” whereas other trained experts were grouped as “formal caregivers.” By understanding the relationship between a user’s role and UI, we can adapt the UI designs to efficiently inform of an older adult’s state. Each UI has a different concept. For example, one UI is designed on a tile-based template, whereas another UI uses a 3D map to present data within its virtual space. The last UI overlays AR contents around a user’s face, and the data are delivered through AR contents. Although the 3 UIs have distinctive design themes, the data displayed on every UI are almost identical, and the interaction modalities supported on each UI are similar with minor differences. On the basis of the meeting with iVO project participants, we hypothesized that informal caregivers would prefer the tile-based UI with audio-based interaction (eg, voice command inputs and audio outputs) because of the simplicity of data presentation and hands-free property. In contrast, formal caregivers would prefer the map-based UI with touching and reading capabilities because of the different data levels, intuitive data visualization, and ease of use while visiting an older adult’s residence. Meanwhile, we assumed that older adults would prefer the AR-based UI with touching and reading capabilities because of engagement, intuitiveness, and easiness. To consider a practical use case that requires mobility, we implemented and evaluated these 3 UIs on mobile devices. We designed our app to be able to use an IoT platform to receive an older adult’s daily activity data.

In this design study, we made the following contributions in the context of health monitoring of an older adult: (1) we designed and implemented the app with 3 initiative UIs for formal and informal caregivers to support the care of older adults using IoT; (2) we conducted user evaluations to analyze user experience and user acceptance of the app and its UIs to identify the relationship between the user’s role and their acceptance of a particular UI, and this would emphasize the necessity for diversity in interaction modalities and UIs; and (3) we observed overall positive user acceptance of using AR by participants and especially among the older adult participants, along with ideas on how AR can be used further in the context of older adult health care.

The design of our system and app is described in detail in the following section. Next, we describe the user evaluation procedure and the data analysis. Then, we present the results of the data analysis, categorized as overall impression and user acceptance, to show participants’ impressions of our app. This paper ends with a discussion of our design study’s implications for the health care monitoring domain and its contributions to future studies.

## Methods

### Development

This section explains the system environment that was used to collect human behavioral data in people’s residences. We then present the design process to build the UIs along with the target device for running our app.

#### App Environment

**Figure 1:**
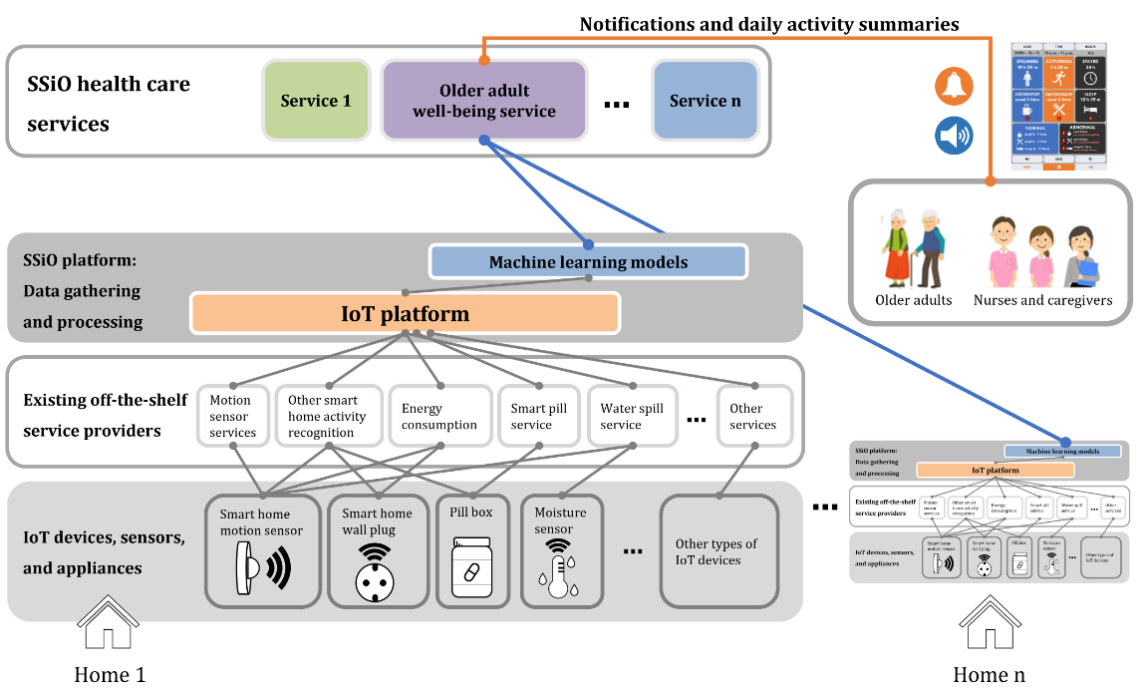
Framework for Societal Development Through Secure Internet of Things and Open Data (SSiO) health care services. IoT: Internet of Things.

In this study, we used the IoT platform Societal Development Through Secure IoT and Open Data for monitoring a person’s daily activities, as shown in Figure 1.

Figure 1 shows the IoT platform being used where arbitrary sensors can be connected; data are gathered, stored, and processed to identify activity in homes. The service designed, developed, and evaluated is the older adult well-being service in Figure 1. Shahid et al [14] give more details on data processing and analytics that designed a framework for preprocessing and processing the data and activity recognition models based on data from the off-the-shelf sensors and IoT devices installed in homes to learn daily patterns of different activities and detect anomalies.

This study aimed to evaluate participants’ user experience and impression of the UIs. The work done in the iVO project [13] also forms the basis for the need to design our app and its UIs as, in that study, information was shared via SMS text message notifications. However, during repeated interviews and communications with the participants and their caregivers, a need for an app with more detailed information to view was observed. The primary data used for visualization in this study were (1) duration of being active (ie, activeness), (2) duration of being still (ie, stillness), (3) duration of staying in a room (ie, as both active and still), and (4) transition logs from one room to others. In addition, Textbox 1 describes all the data used to detect activity in each room.

**Figure 2:**
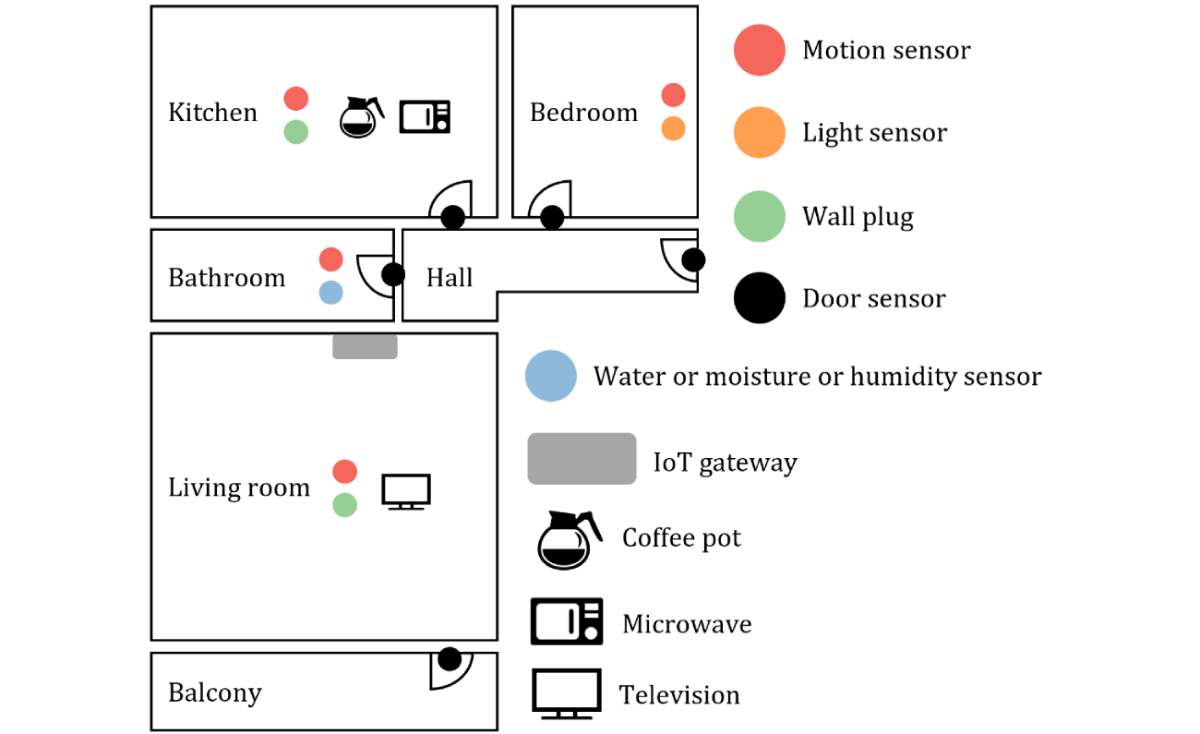
Sensor installations in an older adult’s residence for collecting daily activity data. IoT: Internet of Things.

Figure 2 illustrates an example of IoT sensor installations in an older adult’s residence. We installed nonintrusive IoT sensors in each room, and the actual sensor installations were adjusted to the room design and available appliances in the older adults’ houses. The app designed for this study could be used to check for both normal and abnormal activities.

The real-world behavioral data collected through the iVO project [13] were used to create a generic older adult’s 3-day behavioral activity pattern and used as sample data for our app.

**Example of collected and processed data for abnormal activity detection.**

- Bathroom
  Duration of stay and number of visits during sleeping time
- Bedroom
  Duration of sleep
- Living room
  Duration of stay and television use
- Kitchen
  Duration of stay and number of appliances used
- Balcony
  Nighttime visits

#### App Design

##### Overview

On the basis of the design principles [20-22], we designed a prototype app that consisted of 3 UIs through several iterations. As we wanted to identify various useful design elements and collect diverse feedback regarding UI design, we prepared 3 different UIs with unique concepts. Once the core features of each UI were implemented, such as tiles with large icons and text, a 3D rotatable map, and floating AR contents around a user’s face, we performed user tests with our colleagues to identify possible improvements in user experience and usability perspectives. We updated the visibility and readability of information on each UI, including an aligned menu design for better accessibility with an intuitive navigation procedure. The following sections describe the interaction modalities and UIs used in the user evaluations.

##### Design Principles

The target user group in our study included older adults in particular; thus, the UI design should consider the age-related elements that could affect the user experience [20-22], for example, big font size and high graphic clarity for visual elements; low-frequency perception and additional stimulation, such as a vibration of a mobile device, for auditory interaction; and a rule-based color theme and simplified menu navigation for cognitive processes. We used these elements as fundamental design principles for our 3 UIs.

For one of the UIs, we used a tile-based design along with text and pictograms inspired by commercial apps such as the Oura Ring [23] and Apple’s home app [24]. We expected that the strength of the tile-based design would be the simplified data presentation with intuitiveness.

**Figure 3:**
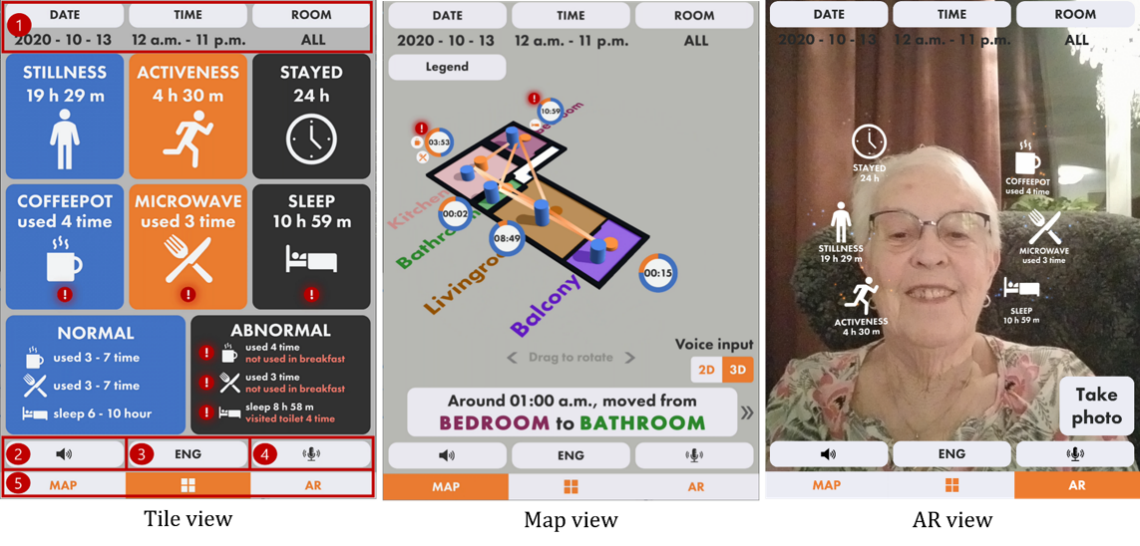
The 3 user interfaces display similar information in different formats. AR: augmented reality.

In the map-based UI, we used a 3D map and several graphical elements on that map to present information. The information presented in all 3 UIs (Figure 3), including the AR-based UI, was similar; however, we found that data presented on a virtual map that refers to a real-world space could further improve the intuitiveness of the information [15,25].

According to our literature survey, the properties of AR have positive effects such as motivation and intuitive data visualization [12,15,26]; therefore, we decided to use these effects in our app to support older adults. Although some studies in the health care domain relied on a printed marker [9,15], we decided to use a face filter style of UI for AR in the older adult health monitoring service to evaluate the acceptance level of AR by older adults.

##### Interaction Modality

We used multimodal interaction to provide flexibility in the UI for older adults [20,21]. However, simply increasing the number of interaction modalities poses a potential failure to achieve effective multimodal interaction [27,28]. Therefore, we used basic interaction modalities that modern mobile devices support instead of adding more modalities using external devices. For example, we enabled touch and voice command and used facial parts as visual cues for input modalities. In contrast, visual elements, sound, and a device’s vibration were used as visual, audio, and haptic output, respectively. The mobile device vibrated when the user pressed a button that contained an abnormal event or an animation to play. Therefore, the vibration was an additional modality to emphasize a notification rather than the principal channel for delivering information, such as visual elements and sounds. We enabled every interaction modality in all UIs as we wanted to evaluate the end users’ initial impression of our app and its UIs that were similar to the final product.

##### UI Design

We designed 3 UIs that present similar information but in different forms. Our primary UI design principle was to achieve a proper level of intuitiveness for reducing the cognitive process of finding and understanding the information. The reason for choosing design principles was that our app’s target user group was older adults, for whom the intuitiveness of data presentation is an essential factor. Figure 3 illustrates our 3 UIs to aid health care tasks performed by formal and informal caregivers of older adults.

We referred to several findings from related studies regarding the UI design for older adults in our app’s UI design, for example, a large font, button, and image size for better visibility; consistent color scheme to increase the readability of the information; simplified menu navigation for ease of use and fast data access; support for offline accessibility to prevent user experience interruption; and simplification of data visualization for intuitive information delivery [20,22,29].

To ensure consistency between UIs, all 3 UIs have 5 shared features marked in the tile view in Figure 3. First, the top 3 buttons are for setting the window to select data. The date button is used for choosing the date. The time button is used for selecting the time window. The room button decides which room data the user wants to see. Second, the speaker button is used to play the audio for reading out the information. When the audio is playing, pressing this button stops the audio. Third, switching the language between English and Swedish is done by pressing the language button. Fourth, the microphone button enables the voice command feature for interacting with the app using a human voice. The voice command consists of 3 keywords to correctly configure the system for receiving data: date, time, and room name. Finally, the bottom 3 buttons are for switching between UIs.

Each UI has a unique design concept for presenting information to users in addition to these common features. We designed the tile view and the map view to provide as much data as possible, from overview to detail, to formal and informal caregivers. In contrast, the AR view was designed for older adults. We decided to present minimum data in the AR view based on interviews with older adults [14,30]. We found that older adults tend not to show interest in the detailed report of their daily activity; therefore, we simplified both the level of data and the visualization complexity.

**Figure 4:**
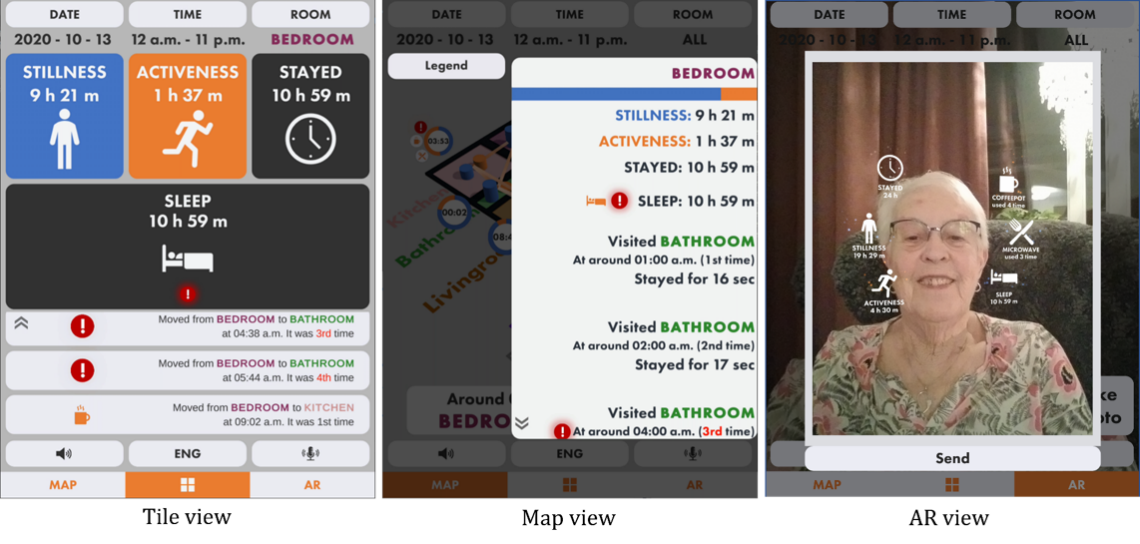
The user interfaces display a transition log between rooms in the tile view and map view. In the augmented reality (AR) view, a feature captures the screen image and AR information for sharing with other users.

First, the tile-based UI that uses rounded squares with large icons with a minimum amount of text to describe the information is named tile view (Figure 3). When abnormal behavior is detected, a correlated tile displays the exclamation mark icon to emphasize that the user has to be aware of it. Each tile is clickable, and the information regarding the pressed tile is played as an audio output. In addition, the device vibrates when the tile with an exclamation mark icon is pressed. A transition log from the selected room to others is presented when a specific room is selected, such as in the tile view in Figure 4.

Second, the 3D map–based UI presenting a person’s behavior data on a 3D-modeled floor plan is named map view (Figure 3). The 3D map is an actual floor plan of the user’s residence, thereby expected to improve the UI’s intuitiveness. The circle icon with a progress bar indicates the percentage ratio of activeness and stillness of a person in each room. The cylinder in a room also represents the activeness and stillness of a person through the cylinder’s height. When an event such as kitchen appliance use or abnormal behavior occurs, additional icons are visible next to the circle icon. For example, when the coffee pot is used during lunchtime, a coffee pot icon is displayed. If not, an exclamation mark icon is visible to represent that abnormal behavior is detected. The map can be rotated by dragging it with a finger, and the view on the map is changeable from perspective view to top view and vice versa. The transition log ordered by time is listed below the map. A correlated trajectory line on the map is animated to highlight the information when the user clicks on one of the buttons on the log list. In addition, the information related to the selected log is played as an audio while the trajectory line is animated. The buttons on the log list can have an exclamation mark icon when the log contains abnormal behavior. In this case, the device vibrates once the user presses the button. The transition log is also provided in another panel depicted in the map view in Figure 4 when the user selects the circle icon on the map. An additional pop-up window appears to show detailed information about abnormal behavior when the user clicks on the exclamation mark icon on this panel.

Last, we used the ARCore (Google) face-tracking feature [31] to use the user’s face as a marker for AR (see the AR view in Figure 3). The data are presented as AR text with AR icons floating around the user’s face. As a result, the user does not need to prepare a printed marker to visualize AR objects. The AR icons are clickable. Once the AR icon is pressed, correlated information is played through audio, and the device vibrates as well. Although the tile view and map view are designed for both formal and informal caregivers, the AR view is designed for older adults. We foresaw that older adults could accept the AR view for the following reasons. First, we minimized the information given in the AR view by focusing on the main activities in each room. Second, AR objects would make older adults engage in using the AR view. Third, the user could capture an image of their face along with data visualized through AR objects. The AR view in Figure 4 shows the captured image with data as AR objects. This captured image could be shared with formal and informal caregivers to inform of the user’s latest state.

The flowchart and user flow of each UI are provided in Multimedia Appendix 1 and Multimedia Appendix 2, respectively. In addition, the summary of each UI’s details, including target user, interaction modalities, and unique features, is presented in Multimedia Appendix 3.

##### Target Device and Configuration

We used Samsung Galaxy Tab S3 tablets with 4-GB RAM and a 9.68-inch screen with 2048 × 1536 resolution on the Android operating system version 9 to test our app. We used Unity (Unity Technologies) to develop the app and used Google’s speech service to enable speech recognition and text to speech in both English and Swedish. Moreover, we downloaded an English-language package for Google’s speech service to make the speech recognition system work with English commands even when the device is offline.

### User Evaluations

#### Participants

We recruited some participants from the study by Shahid et al [14]. They voluntarily joined our user evaluation. Furthermore, we approached more older adults in Skellefteå, Sweden, with similar profiles as those in the iVO project. We tried to recruit people in three different roles: (1) older adult, (2) formal caregiver, and (3) informal caregiver.

#### Experimental Procedure

##### Overview

For our study, we designed the user evaluation test to run for 1 hour for each participant. This involved 30 minutes of firsthand experience using our developed app and its different UIs followed by an interview for 20 minutes. During the user evaluation, the participants freely navigated each UI, and a researcher assisted them in experiencing every feature of our app. Finally, the participants were handed a questionnaire to fill in on their own, which took approximately 10 minutes. During the evaluation, the participants interacted with the app keeping in mind their personal context of being a formal or informal caregiver to an older adult or being an older adult using such an app for themselves.

##### User Evaluation

Owing to the pandemic, we were limited in meeting participants from many nursing home and caregiving domains. As a result of the social distance policy, we met participants with up to 4 people at once. When we arranged a meeting with an older adult, we always grouped them with their informal caregivers or friends to make the older adult feel comfortable during the evaluation. Before starting the evaluation, we informed each participant about the process and obtained their consent. The participants were free to withdraw if they felt uncomfortable. In the user evaluation, we explained our app and the features of the UIs while the participant had firsthand experience with them. We introduced each UI in the following order to emphasize the difference between them: (1) tile view, (2) map view, and (3) AR view.

##### Individual Interview

After the participant had finished experiencing all the app features, we conducted an individual interview. During the interview, the conversation between participants and researchers was recorded under agreement for data analysis later. A number of questions were designed by referring to the technology acceptance model for the interview [17]. We asked about their impression and perception of the UIs and app features throughout the interview (eg, which UI was preferred based on the purpose of app use, which interaction modality helped use the preferred UI, and how easy to use and useful were those UIs and interaction modalities). We chose certain questions according to the conversation during the interviews with participants to allow for flexibility. Multimedia Appendix 4 provides a full list of interview questions.

##### Questionnaire

We used the User Experience Questionnaire (UEQ) designed by Laugwitz et al [32] to evaluate overall impression of the app in terms of usability and user experience. According to Laugwitz et al [32], the usability aspect comprises “efficiency,” “perspicuity,” and “dependability,” whereas the user experience aspect includes “novelty” and “stimulation.” The original UEQ contains another scale named “attractiveness” measuring another aspect of impression of the app using 6 items (ie, “annoying/enjoyable,” “bad/good,” “unlikable/pleasing,” “unpleasant/pleasant,” “unattractive/attractive,” and “unfriendly/friendly”). We omitted the attractiveness scale in our questionnaire as we were only interested in usability and user experience. As a result, we included only 5 scales (ie, “efficiency,” “perspicuity,” “dependability,” “stimulation,” and “novelty”) with 20 items in the questionnaire. The efficiency, perspicuity, and dependability scales represented pragmatic quality aspects (ie, task-related) related to usability. In contrast, the stimulation and novelty scales comprised hedonic quality aspects (ie, non–task-related) related to user experience.

In the questionnaire, general information was asked about a person’s gender and age in a range. Then, 20 items were given to be answered with a 7-stage scale. Each item contained 2 opposite words, and a participant had to select a stage representing the closest scale between 2 words. The order of the words was randomized, and the order of positive and negative words was also shuffled for each item to make the participant focus on reading each item instead of selecting words with a consistent pattern. Groups of items in the same scale had similar meanings to ensure consistency in a participant’s answer. In other words, a participant’s answer could be unreliable when inconsistency arose.

### Ethics Approval

This study was based on the iVO project conducted by Shahid et al [14]. The participants consented to the collection and recording of their questionnaire answers and interview data during the user evaluations. The project was, overall, in compliance with the European Union General Data Protection Regulation guidelines [33]. The data collection and processing in this study were approved by the Regional Ethical Board in Umea, Sweden (diary 2018-189/31).

### Data Analysis

We conducted a statistical analysis of the questionnaire answers to identify the potential end users’ overall impression of our app. To evaluate the user experience of the 3 UIs from the questionnaire answers, we used an analysis tool provided by the UEQ team [34,35]. The analysis tool calculates means, SDs, and CIs per item and scale. The margin of error at a 95% CI was calculated by using the *t* value because of the sample size (N<30). In addition, a comparison of the results with those of other studies evaluated using the UEQ is presented as a benchmark. The interview answers were coded [36] to identify common impressions of participants on the 3 UIs and interaction modalities. We used inductive coding to organize data generated from observations of participants and interviews.

## Results

### Overview

**Figure 5:**
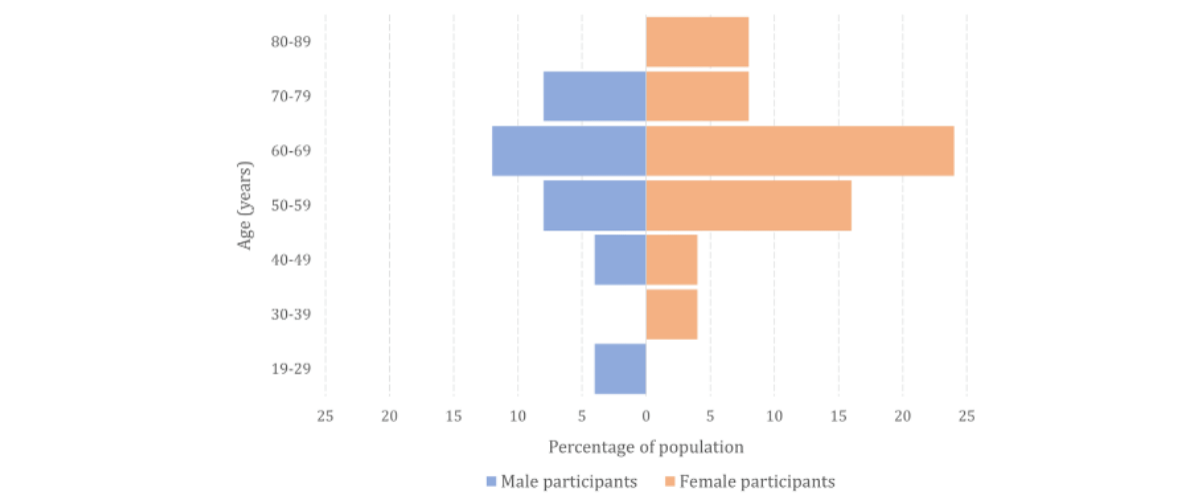
The participant population by age group.

As participants in this study were from the study by Shahid et al [14], they all had experience using a health monitoring system. In the end, we had 26 participants—17 (65%) female and 9 (35%) male. We met 96% (25/26) of participants in person, whereas we met 4% (1/26) on the web because of the limited contact owing to his job specialty during the pandemic. We removed 4% (1/26) of participants (P20) from the quantitative data because of the inconsistency in her questionnaire answers. The UEQ was used to measure the overall impression of our app, and the interviews were conducted to obtain qualitative data that could be used to understand user acceptance of the UIs and interaction modalities based on the user’s role. We categorized participants into three groups based on their role instead of their age: (1) older adult, (2) formal caregiver, and (3) informal caregiver; of the 26 participants, there were 12 (46%) older adults, 1 (4%) formal caregiver, and 13 (50%) informal caregivers. Apart from the participant whose job was as a formal caregiver, 2 ( 8%) participants from medical services, a nurse (P17) and a physician (P23), attended the evaluation. Most participants in the informal caregiver group (6/13, 46%) were aged from 50 to 59 years, whereas most participants in the older adult group (6/12, 50%) were aged from 60 to 69 years. All older adults (12/12, 100%) were aged >60 years, and the formal caregiver was in his 20s. Table 1 shows the information of each participant, and Figure 5 shows the demographics of the participants. The collected questionnaire data were normally distributed, as we could verify from quantile-quantile plots of the means of each scale per participant (Multimedia Appendix 5).

**Table 1:** Information about the 26 participants in 3 roles: older adult, formal caregiver, and informal caregiver.

ID	Gender	Age (years)	Role
P1	Woman	60-69	Informal caregiver
P2	Woman	80-89	Older adult
P3	Woman	60-69	Informal caregiver
P4	Woman	50-59	Informal caregiver
P5	Man	19-29	Formal caregiver
P6	Man	50-59	Informal caregiver
P7	Woman	30-39	Informal caregiver
P8	Woman	70-79	Informal caregiver
P9	Woman	50-59	Informal caregiver
P10	Man	70-79	Older adult
P11	Woman	80-89	Older adult
P12	Woman	70-79	Older adult
P13	Woman	60-69	Older adult
P14	Woman	60-69	Older adult
P15	Man	60-69	Older adult
P16	Man	70-79	Older adult
P17	Woman	50-59	Informal caregiver
P18	Woman	50-59	Informal caregiver
P19	Man	40-49	Informal caregiver
P20	Woman	70-79	Older adult
P21	Woman	40-49	Informal caregiver
P22	Woman	60-69	Older adult
P23	Man	60-69	Informal caregiver
P24	Man	60-69	Older adult
P25	Woman	60-69	Older adult
P26	Man	50-59	Informal caregiver

### Overall Impression

#### Overview

We quantified 20 quality aspects that consisted of 2 words for each item in the questionnaire. We analyzed and benchmarked the responses based on the UEQ scales [34,35]. We also listed overall impressions of our app identified from the interviews.

#### UEQ Results

##### Quantified Quality Aspects

We calculated the mean, SD, and CI of each item in the UEQ that was transformed from the 7-stage scale into −3 to 3 values to evaluate the quantified quality aspects of both user experience and usability of our app. The results of each item are provided in Multimedia Appendix 6. Each item’s SD and CI were calculated from the mean of participants’ answers to each item. A total of 95% (19/20) of the items were answered over a mean of 1.6 (SD 1.19), whereas 5% (1/20) of the items (ie, “unpredictable/predictable”) were answered with a mean of 0.60 (SD 1.26). However, as the SDs for 2 items (ie, “cluttered/organized” and “confusing/clear”) were similar to their means, the differences between means and SDs were relatively smaller than for other items. Hence, we have difficulty simply accepting the results of these items as positive. In particular, “unpredictable/predictable” showed the lowest mean among all items that entered the neutral evaluation area. On the basis of CIs, some items’ results were acceptable as a positive evaluation even though they had a high SD. For example, the CI ranges for “cluttered/organized” (ie, 95% CI 0.88-2.32) and “confusing/clear” (95% CI 1.09-2.59) were >0.8, which is the minimum value for a positive evaluation, whereas those items’ means were >0.8 as well.

##### Scale

**Figure 6:**
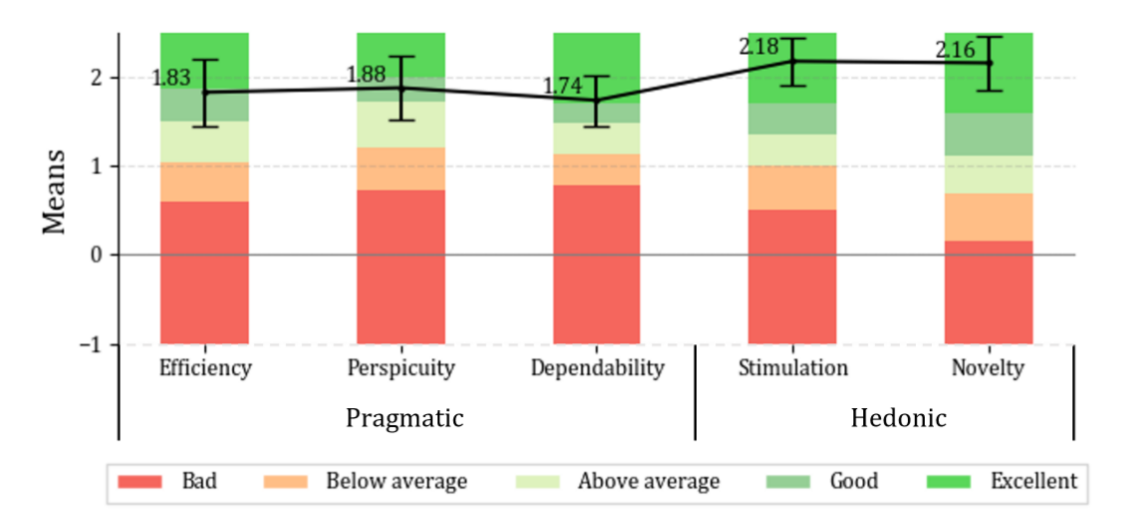
The mean and CI of each scale are depicted with black dots connected with lines on top of the benchmarks of each scale’s mean value, calculated from 21,175 persons in 468 studies published until 2021. The CI of each scale is calculated from the mean of each participant for each scale.

The mean with CI error bars for each scale is shown in Figure 6. Unlike the CIs in Multimedia Appendix 6, the CIs of each scale in Figure 6 were calculated from each participant’s mean for each scale. All scales showed a positive evaluation with a mean >1.74, and stimulation was the most valued scale. This provides evidence of positive evaluations regarding usability in terms of efficiency, perspicuity, dependability, stimulation, and novelty. When each scale was grouped into the quality aspect, the pragmatic quality aspect had a mean of 1.82, and the hedonic quality aspect had a mean of 2.17. These results represent that overall user experience in terms of task- (ie, pragmatic) and non–task (ie, hedonic)-related quality aspects received positive evaluations.

##### Benchmark

The UEQ team have summarized the results of UEQ from a total of 468 other researchers’ studies who also used the UEQ analysis tool. This benchmark was established from 21,175 persons’ data and is illustrated in Figure 6, along with the UEQ results for our app. We found that 2 scales (ie, “efficiency” and “perspicuity”) were rated as the second-best quality (ie, “Good”) and 3 scales (ie, “dependability,” “stimulation,” and “novelty”) were rated as the top quality (ie, “Excellent”).

#### Interviews

##### Overview

We categorized the participants’ interview data into 2 parts, and each category consisted of the following keywords. The first category contained participants’ feedback on overall impression caused by informative data, intuitive UI design, ease of use, age dependency, and lack of design clarity. The second category included user acceptance regarding the UIs and interaction modalities based on a level of ease of use and usefulness, which is presented in a separate *User Acceptance* section. Some interview answers that were notable for understanding participants’ perceptions of our app, UIs, and interaction modalities are provided in Multimedia Appendix 7.

##### Informative Data

Participants experienced that the data were informative to understand a person’s state. For example, an informal caregiver (P8) showed interest in the map view because of the supportive information for monitoring an older adult. Participants also experienced that the data on the UIs were supportive of care in a case where an informal caregiver had a problem obtaining necessary information while meeting her parent. A similar opinion was expressed by one of the informal caregivers:

Even a small event like visiting a toilet can be checked that my parent may not remember anymore.

P1

Furthermore, another informal caregiver (P18) imagined how valuable the data could be to overcome the time and distance issues that prevented her from knowing her parent’s condition. Older adults evaluated the data as positive because of the beneficial outcomes for formal and informal caregivers. For example, an older adult (P13) thought about how useful the data could be in a specific scenario, such as when an older adult has cognitive impairment:

I can feel safer if I have this. Someone knows that I am still moving around. For instance, my children can see that I am moving. If you develop dementia, perhaps, you don’t know if you've eaten or not. This can tell if you did it.

P13

One of the benefits of obtaining data for informal caregivers is that it helps understand the older adults’ states before visiting their residences (P19). The formal caregiver (P5) found that obtaining data through the tile view was preferable for him in terms of data acquisition speed and high readability.

##### Intuitive UI Design

Regarding the UI design, participants experienced the intuitiveness of the UIs for acquiring data. Several graphical elements were identified as helpful visual cues to aid participants in understanding the data. In the map view, the icons on buttons and the cylinders in each 3D room were perceived positively. In addition, the data visualization on the 3D map helped understand the data with spatial cues. We explained to participants that the 3D map would be the map of their residences. The data were presented in the corresponding room in the 3D map. As a result, participants experienced that the data presentation based on data-related room positions leveraged intuitiveness. For example, participants stated the reason for choosing the map view as it being a better UI than others (P10 and P11).

In the tile view, the color theme was positively received because of the improved visibility and readability of the data. For example, an older adult (P14) liked the color theme as she could obtain data by skimming through the color on each tile. When she saw the red icon on a tile, she could become aware of which activity had an abnormal behavior history before reading detailed information written in text. Different colors used for each purpose aided her in understanding the data in a short time. In addition to the design elements, the formal caregiver (P5) noted the simplicity and intuitiveness of the tile view’s layout. He found the tile view to increase the usability of the app for a caregiving service owing to quick and easy data access.

##### Ease of Use

Some of the participants (5/25, 20%) admitted that time was needed to get used to our app; however, 68% (17/25) of the participants explicitly mentioned how easy it was to use our app. We found these participants from all age groups and in every role. The individual preferences for UIs are unique to each participant; however, they all experienced the easiness of data acquisition.

##### Age Dependency

Participants felt that, even though our app was easy to use, their parents would require more time to get used to using it because of their unfamiliarity with a smartphone and app. One of the informal caregivers (P7) pointed out the different levels of user acceptance between the younger and older generation by adding an extra element, that is, a “skill,” which can be called “familiarity,” established by previous experience:

[This app is] suitable depending on the user...Not only the age but also the skills that the user has affected the experience. The younger generation can enthusiastically use it.

P7

As evidence, we observed in the user evaluation that a relatively young adult could learn how to use AR much faster and explain it to their parent, who took more time to be able to use it by themselves. In addition, the formal caregiver (P5) showed a pessimistic perspective on the user acceptance of especially AR by older adults for the same reason that others expressed: unfamiliarity.

##### Lack of Design Clarity

Despite the positive experience that the app provides, some participants (6/25, 24%) experienced inconvenience from UIs caused by (1) the ambiguity of data visualization in the map view, (2) the vague motivation for use, and (3) the lack of consideration for user experience in UI design.

### User Acceptance

#### UI Acceptance

##### Overview

**Figure 7:**
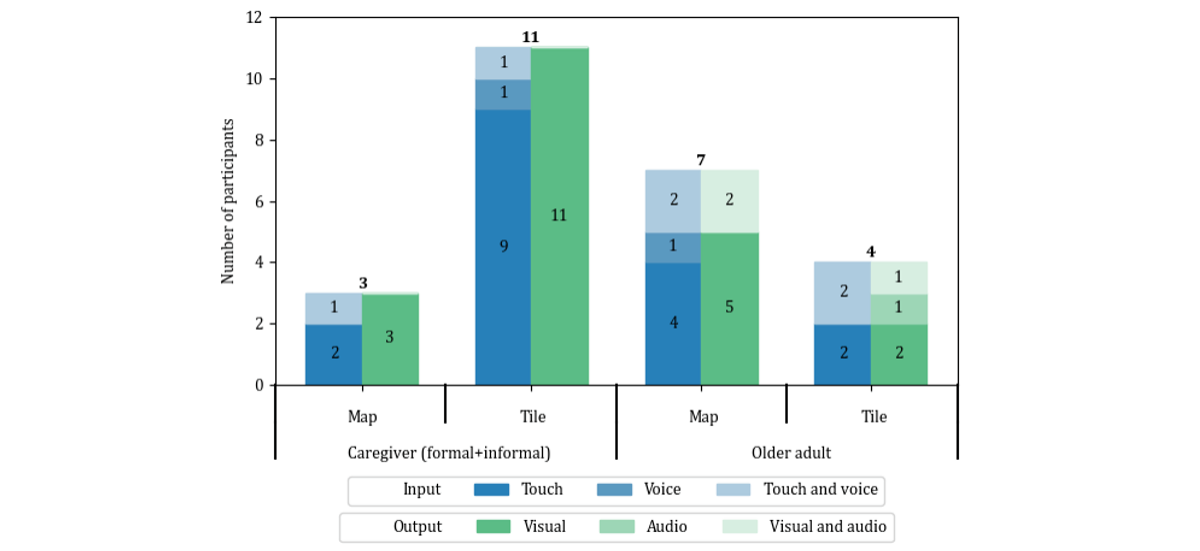
The role-based user preference for the interaction modalities in 2 user interfaces.

We analyzed the participants’ UI preferences grouped by the user’s role: (1) the caregiver (ie, formal and informal) and (2) the older adult. Figure 7 illustrates the user preference for interaction modalities in the map and tile views depending on the user’s role. Personal preference could be owing to various reasons; hence, we focused on the reasons for choosing a specific UI in terms of ease of use and usefulness. Table 2 summarizes the reasons for UI preference by participant role.

**Table 2:** Summary of reasons for user interface (UI) preference by each participant role: older adult (O), formal caregiver (F), and informal caregiver (I).

UI and role			Reason for preference
**Map**			
	O	Data presentation in a correlated room in the 3D map Visual representation of an older adult’s movements with trajectory lines Support for different levels of depth for data presentation	
	F^a^	Data presentation tool in a meeting with others because of visual graphic components	
	I	More intuitive than other UIs because of the visual graphic components Overview of daily activity instead of detailed data	
**Tile**			
	O	Simple UI design for easily understanding the overview of data	
	F	Simple UI design with informative data without unnecessary information	
	I	Simple UI design for fast data acquisition Familiar UI design More detailed data than in the map view	
**AR^b^**			
	O^a^	Communication with others for social interaction As a condition report in an emergency to provide additional data	
	F^a^	Active participation in health care rather than being observed	
	I^a^	Additional data collection, such as facial expressions Making older adults participate in health care Feeling relieved through communicating with others	

^a^Impression rather than a reason for preference.

^b^AR: augmented reality.

##### Map View

The map view was the second most preferred UI among participants, chosen by 40% (10/25). The intuitiveness of the map was the reason that participants in all roles selected it as their preferred UI. In total, 58% (7/12) of the older adults preferred the map view for mainly three reasons: (1) the data were placed in related rooms, (2) the movement lines were visually presented, and (3) the data were available in both overview (see the map view in Figure 3) and detailed (see the map view in Figure 4) views. The formal caregiver proposed an idea to use the map view as a way to inform and communicate details about patients (ie, older adults) to clients (ie, informal caregivers). In total, 23% (3/13) of the informal caregivers wanted to see an overview of daily activity. Visual elements such as icons and cylinders on the 3D map helped them understand an older adult’s state in a short time.

##### Tile View

The tile view was the most preferred UI, chosen by 60% (15/25) of the participants. The principal reason for preferring the tile view was the intuitive UI design. Regarding the UI design’s intuitiveness, not only a simple UI design but also a familiar UI design could be perceived as an intuitive interface [37]. Participants in every role perceived the tile view as an intuitive and effective UI for overviewing data because of the simple design. Older adults wanted an overview of the data, and the formal caregiver preferred an overview with less detail, which was unnecessary for him. In addition, informal caregivers expressed that the tile view provided more detailed data than the map view, and they got used to the tile view because of the similar design to the app they had used before.

##### AR View

None of the participants selected the AR view as their preferred UI; however, most participants (10/14, 71% of caregivers and 10/11, 91% of older adults) showed interest in using the AR view as a supplementary tool for additional data mining and social interaction. Informal caregivers claimed that their parents’ facial expressions gave additional information not written in the text. Furthermore, other informal caregivers perceived that using the AR view could make them feel relieved by communicating via a facial image and activity data. Meanwhile, the older adults had a positive impression of using the AR view to communicate with their children. Sharing the captured facial image and conversing about it with others would amuse older adults who might be lonely. In contrast, sharing the captured image was perceived as a visual report for older adults to update their families on their condition. The formal caregiver declined to use the AR view; however, he saw potential use by older adults within a health care service, as did an informal caregiver (P9), because of the active participation of the older adults in their health care rather than being passively observed by others.

#### Interaction Modality

##### Overview

Our app supports multiple interaction modalities. This section analyzes the participants’ preferences for the input and output modalities. The summary of reasons for interaction modality preference is shown in Table 3. Similar to the reasons for the UI preferences, personal preferences on interaction modality could vary; hence, we focused on the reasons in terms of ease of use and usefulness. We speculate on several participants when they did not explicitly express the reason for modality preference. As the vision as an input modality in the AR view and the vibration as an output modality in every view were not principal modalities for delivering information, we excluded them from Table 3.

**Table 3:** Summary of reasons for input and output interaction modality preference by participant role: older adult (O), formal caregiver (F), and informal caregiver (I).

Interaction modality and role			Reason for preference
**Input**			
	**Touch**		
		O	Familiar modality
		F^a^	Touch is faster than voice command
		I	Comfort with touching for navigating the UI^b^ because of many buttons Simiple and familiar modality
	**Voice**		
		O^a^	Comfort with giving voice commands for navigating the UI because of ambiguous button designs
		I^c^	Alternative modality for those who need another channel for interaction
	**Touch and voice**		
		O	Switchable modality depends on a user’s state
		I	Find suitable modalities by using each of them
**Output**			
	**Visual**		
		O	Location-based intuitive data presentation Meaning of colors helps understand data
		F	Reading is faster than listening
		I	Quick understanding of data Familiar to read information
	**Audio**		
		O^a^	Comfortable with listening rather than reading data on the screen
		F^c^	Alternative modality for people who want to listen
		I^c^	Different information from that of the written text can be delivered
	**Visual and audio**		
		O	Selectable modality depending on a user’s state

^a^Speculation based on a participant’s feedback and observations.

^b^UI: user interface.

^c^Impression rather than a reason for preference.

##### Input Modality

Finger touches and voice commands were used as input modalities. In addition, we identified some participants who preferred to use both modalities.

Touch input was preferred by 92% (23/25) of the participants, including participants who chose multiple modalities. The principal reason was that participants felt that the touch interaction was simple and familiar on a smartphone. Older adults and informal caregivers remarked on the simplicity and familiarity of touch interaction. Another reason given by one of the informal caregivers (P7) was related to the characteristic of the preferred UI. As the map view has various objects to click on for navigating data, P7 felt comfortable touching them instead of using the voice command that required memorizing every command for proper use. The touch interaction required fewer steps than the voice interaction to obtain the desired data. The formal caregiver emphasized how vital the data acquisition speed was for him. Therefore, we speculate that the formal caregiver preferred touch because of the speed of interaction.

Regarding the voice command, one of the older adults (P10) perceived the map view as better than the tile view for obtaining information. However, unlike P7, he felt that he could better control the map view with voice command in comparison with the touch interaction. Therefore, he preferred the voice command over the touch interaction. In addition, some informal caregivers who preferred touch interaction found the value in voice command as an alternative modality for people who have an obstacle to using touch interaction. P19 noted the following:

If you are blind, I can imagine you have a different perspective [on the value of voice interaction] than I do.

Some participants (6/25, 24%) wished to have both input modalities for mainly 2 reasons. First, a physical impairment caused by aging or an injury changes the modality preference. In total, 33% (4/12) of the older adults, who chose both modalities, admitted that touch interaction would be the primary interaction modality when they started using the app because of its simplicity and familiarity. Meanwhile, 15% (2/13) of the informal caregivers initially wanted both modalities as they needed time to decide on the main modalities. Once they chose specific modalities as their primary interaction, they would like to stick with them.

##### Output Modality

Participants could obtain data through visual elements (eg, icons, text, and 3D objects) and audio. Similar to the input modality, we found that some participants wanted to have both output modalities.

Visual elements as output modality were perceived positively for mainly 3 reasons. The first was intuitive data presentation with locational information. Specifically, the map view used various visual elements such as icons, lines, and cylinders on the 3D map to provide information about a person’s behavioral activities, such as transitions between rooms, overall time spent in a room, and activity in each room. Older adults experienced the strength of the visual elements on the map as they could understand information by simply seeing them instead of reading text. Another older adult (P14) reported the role of colors in recognizing data on the tile view. As long as the visual elements have a simple and understandable design, the data can be successfully delivered to participants in a relatively short time compared with audio output. The speed of data acquisition was the second reason for the preference. The formal caregiver (P5) preferred the tile view rather than the map view, mainly as reading text was fast and convenient for him. Some informal caregivers (2/13, 15%) also liked to see the data on either the map view or the tile view as they could obtain information quickly by seeing visually represented data. The last reason for this preference was familiarity with reading. People are used to reading content; therefore, many informal caregivers chose visual elements as their preferred output modality.

The audio output is the system’s feature to read text when a user presses a button. The system reads either displayed data on the screen or a summary of the data the user is seeing. An older adult (P12) preferred this audio output while using touch interaction. According to P12, even touch interaction was challenging for her; however, it was relatively more manageable than the voice command. Hence, she chose touch interaction as the main input modality. From this, we speculate that her choice of audio as a preferred output modality was made because of the relatively simple process to obtain information compared with reading. An informal caregiver (P6) pointed out that using the audio output had little merit as there was no difference in information between the written text and audio output. In other words, the participants may be willing to use audio output if there is a difference in information between the 2 different outputs. In addition, the formal caregiver (P5) found a potential use case of audio as an alternative output modality for specific users who have a reading disorder or do not want to read.

In total, 25% (3/12) of the older adults answered that they preferred having both output modalities to consider when switching between them. For example, a change in the user’s physical condition caused by aging may trigger the modality change. In other words, they considered using the 2 output modalities separately rather than simultaneously.

## Discussion

### Principal Findings

We used the UEQ to evaluate the initial overall impression of our app in terms of usability and user experience. The questionnaire answers regarding pragmatic (ie, usability) and hedonic (ie, user experience) quality aspects showed that most items from all scales were rated positively. The participants positively evaluated all the items on every scale except the dependability scale. We identified an item (ie, “unpredictable/predictable”) from the questionnaire data analysis with a relatively low mean and high SD compared with other items in the dependability scale. This result implies that there is room for improvement regarding the unpredictable behavior of our app against the user’s expectations. However, there could be another reason for this that needs to be further investigated. This could be the participants’ different understanding of the questionnaire items [35]. This confusion could be caused by the participants’ context while taking the questionnaire. For example, the item “unpredictable/predictable” asked whether our app had reacted as the participants expected. However, several participants (5/25, 20%) asked about the meaning of “unpredictable/predictable.” In addition, we found that some participants who selected negative or neutral words for “unpredictable/predictable” chose positive words for other items on the same scale. Therefore, we assume that this result could be caused by either a misinterpretation of an item or an outlier. The benchmark was used as complementary data to show the quality of our app, and we found that our app was rated as at least “Good” on all scales. However, the mean of the “dependability” scale was relatively lower than that of other scales. As the item (ie, “unpredictable/predictable”) in the “dependability” scale could affect the result, we presume that an evaluation with a clear explanation and additional participants could provide more reliable results. Overall, the participants expressed interest in our app because of its usefulness for checking an older adult’s condition through intuitive UIs and ease of use with a steep learning curve [38]. The questionnaire results support the interview answers. For example, positively rated words such as “supportive,” “valuable,” “motivating,” “easy,” “understandable,” “easy to learn,” and “clear” support the participants’ answers regarding “informative data,” “intuitive UI design,” and “easy to use.”

Although 68% (17/25) of the participants expressed that our app was easy to use, some participants (6/25, 24%) still expressed uncertainty about the UI design in terms of “ambiguous data visualization,” “vague motivation,” and “lack of consideration for user experience in UI design.” In addition, the inconvenience invoked by unfamiliarity was a noticeable phenomenon among older adults. Informal caregivers were concerned about this problem for their inexperienced parents when a new technology was introduced, such as the AR view. To resolve the uncertainty, each UI should be finely designed (1) to provide a clear meaning in visual elements, (2) to stimulate end users with a reasonable and sufficient motivation for feature use, and (3) by giving enough consideration to user experience. In addition, the learning process should be supported with media, such as video demonstrations [39], to help older adults get used to the app and UIs.

Throughout the analysis of user preference for the map and tile view, we identified “intuitiveness” and “simplicity,” the importance of which was verified by other studies [20,40,41], as the factors affecting user acceptance to a greater extent. A total of 64% (7/11) of the older adults preferred the map view as it was intuitive because of various visual elements combined with locational data, whereas the tile view impressed 79% (11/14) of the caregivers with its simple UI design. We then identified that the most preferred input modality by participants in every role was touch interaction as it was simple, fast, and familiar. Even though several older adults and a few caregivers (8/25, 32%) were interested in using the voice command, it was perceived as a secondary rather than a primary modality. Regarding an output modality, all the caregivers (14/14, 100%) liked to see the information because of fast data acquisition. Several older adults (4/11, 36%) wanted to listen, whereas 64% (7/11) still preferred to read the data from visual components.

The results of user preference for the UIs and interaction modalities go against our hypotheses. The identified reason for selecting the tile and map view were as we expected; however, both types of caregivers selected the tile view, whereas older adults were interested in the map view. We hypothesized that formal caregivers would like to use the map view for comprehensive data provided by intuitive visual components. According to the interviews, data acquisition speed was the primary factor for using our app by the formal caregiver. Therefore, obtaining information from the app should be swift and concise. Although the map view could provide fruitful data intuitively, a simple UI for fast reading of information was prioritized. As expected, informal caregivers preferred the tile view. However, as the voice command and audio output had several drawbacks, such as a necessitating learning and being slower than reading visual outputs, informal caregivers highly relied on touch interaction for as input modality and visual elements as output modality. We can mitigate the drawbacks by updating the app to understand natural languages for voice commands. Regarding the older adults’ preferences, we confirmed that touch interaction was the primary modality owing to familiarity. Unlike the caregivers, a relatively higher number of older adults (5/11, 45%) were interested in using the voice command. Output modality preference was also different from that of caregivers in that several older adults (4/11, 36%) wanted to listen because of feeling comfortable with it. Our hypothesis about older adults was incorrect as many older adults showed interest in using the voice command and audio output. Although both output modalities were perceived as secondary, having an available alternative is important because of the possibility that older adults’ state requires another modality for interaction.

The benefit of using multiple modalities is the flexibility of the interaction so that users can decide upon their preferences and states. We expect that the flexibility would enable users to have a better user experience than with a unimodal interaction modality. However, supporting multiple interaction modalities without an apparent purpose is less beneficial than unimodal interaction [27,28]. Similar to the voice command, we identified that the audio and vibration for output modality needed a redesign. As the audio output read aloud almost identical information to that on the screen, participants received the same information again, which was less valuable. To resolve this issue, we can make the audio and written information on the screen different. Essential information should remain the same; however, a slight change in the audio output could be applied for engagement. We also received several comments regarding the vibration. First, it was barely sensible because of the subtle intensity. Second, the icons on the screen already provided information that the vibration tried to notify. As a solution, we can renovate the vibration to enhance the notification with an SMS text message and push alarm. Giving a user the option to configure the amplification and repetition of the vibration can be another improvement.

During the interviews, 100% (1/1) of the formal caregivers and 31% (4/13) of the informal caregivers doubted that their parents would use the AR view. They were concerned about their parents’ low acceptance of the AR view because of unfamiliarity, health-related constraints, and complex procedures. Indeed, AR is not a familiar technology for older adults who are not even familiar with a smartphone. As the formal and informal caregivers predicted, none of the older adults chose the AR view as their preferred UI. However, 10 older adults (n=4, 40% aged 70 years and n=6, 60% aged between 60 and 69 years) perceived the AR view as acceptable to use. Overall, 84% (21/25) of the participants perceived the AR view positively, which was contrary to several caregivers’ assumptions. On the basis of the positive user acceptance of the AR view by participants aged >60 years, we presume that relatively younger generations will be more open-minded about using AR when they get older as they are familiarized with AR apps that are widely popularized, such as Facebook, Instagram, and Snapchat. In fact, we identified that 77% (10/13) of the informal caregivers perceived the AR view as useful, and the principal reason for showing interest in using the AR view among them was the informative aspect of the facial image. Informal caregivers expressed that checking their parents’ faces and reading the activity information helped them seek clues about symptoms that showed in their facial expressions. They also commented that seeing their parents’ behavior data while talking to them would be more convenient than them sending an image. Accordingly, we anticipate positive feedback on enabling the AR view during a video call, which needs further study.

### Limitations

The number of participants per user role in our study was 14 caregivers (n=13, 93% informal and n=1, 7% formal) and 11 older adults. However, the participants in each user group were homogeneous in terms of having experience with health care services. Therefore, the results of the qualitative interview analysis in each user group, especially the older adult and informal caregiver groups, were saturated with an acceptable level according to criteria from other studies [42,43]. Furthermore, the results of the overall impression of our app were reliable as an initial end-user evaluation as we recruited >20 participants of various age groups, of different genders, and in diverse roles [44,45]. In general, during the COVID-19 pandemic, we had difficulty recruiting participants for the user evaluation. In a future study, we will recruit more participants to improve the reliability of the results. In the context of measuring credibility, our participants had 1 hour to experience and evaluate our app’s design. This time constraint may have hindered the participants from having enough time to try every feature of our app in a real use-case scenario. In addition, as we aimed to evaluate initial impressions, we conducted the user evaluation without a task for measuring task-related performance. In the future, a long-term evaluation can be conducted to collect data in real life to identify the issues that influence user acceptance. This evaluation will enable us to measure the perceived usefulness through practical tasks in real life.

Our app shares the personal data of an older adult with caregivers; thus, data privacy concerns are inevitable. A participant raised an important point about a potential violation of personal privacy. Such potential conflicts regarding privacy and security can be mitigated by allowing the end user to decide what data can be shared and establishing different security layers to prevent unauthorized users from accessing the data.

### Comparison With Prior Work

Health monitoring systems have been widely studied as various IoT sensors enable a system to read the contextual information of an older adult [46,47]. The objective of a monitoring system is to understand the states of persons, environments, and products based on the collected information; thereby, a service that is useful for an older adult and formal and informal caregivers could be delivered. It could be a service to aid an older adult’s daily life by providing information [48,49] or services to detect abnormal events in an older adult’s activities to inform a formal or informal caregiver [50,51]. As health care services require a number of technologies to run, user acceptance of the technologies used should be evaluated to validate their effectiveness. We found a few studies that conducted user acceptance testing on health care services; however, the target user groups were young people [52,53] rather than older adults. Moreover, other studies aimed at older adults used a stationary device at a nonindividual residence [54] or used 2D visual components only [55].

To perceive an older adult’s state precisely, it is favorable to use as many data types as possible instead of a single data type because of the different levels of richness of the identifiable information. For example, a passive infrared motion sensor could identify a person’s presence in a place; however, information from biosignals that are useful to understand a person’s physical and mental states could not be identified [56]. Pinto et al [57] even used several types of data, such as an accelerometer, room temperature, and body temperature, to track a person’s states; however, the necessity of additional sensors for collecting a person’s vital signals to monitor in-depth body conditions was stated as future work. In fact, researchers have attempted to use different types of IoT sensors to gather various types of data to understand a person’s state in detail with reliable accuracy [58,59]. The activities of daily living [60,61] and a person’s physical state [62,63] are examples of data that health monitoring systems use. Furthermore, with the growing scale of data quantity and the increasing data complexity, the data analysis method is shifting to use machine learning for improving system performance and handling large-scale data effectively [64-68]. The advantages of data diversity and machine learning adaptation in a smart health monitoring system are decent. Moreover, we found similar advantages of using multiple data with machine learning in the study by Shahid et al [14]. In the study by Shahid et al [14], various indoor sensors such as door, motion, and power plug sensors were used to collect data, whereas specific sensor data were used in the algorithm that was designed to track an older adult’s daily activities with reliable accuracy. When abnormal behavior was detected, an SMS text message was sent to a resident’s formal or informal caregiver to inform of the abnormality.

However, we wanted to go a step further than SMS text message notification for delivering information to users, including older adults and their formal and informal caregivers. As there was an explicit need for an app communicated by participants throughout the iVO project, SMS text message notification was used only for notifying abnormal events; however, our app can highlight or visualize different aspects of the older adult’s activities in detail. Well-designed data visualization could help a user understand information more quickly and easily, thereby expanding the data accessibility to those who might have an obstacle to using such a mobile health care app. Accordingly, we developed the UIs, the map view, tile view, and AR view, for our app based on the data from the study by Shahid et al [14].

Regarding the user experience, several researchers have evaluated AR on different devices, such as smartphones [9,69], tablets [26], a projector [10], and head-mounted displays (HMDs) [11,12,70], to find a beneficial aspect of using it in health care. Using an HMD sounds promising for AR apps as a camera on an HMD is always available. In contrast, other devices require extra effort, such as holding a smartphone to view and installing a camera that links to a projector for motion capture. However, we chose tablets as a target device for running our app for the following reasons. First, HMDs are uncommon in a house where an older adult lives alone. Second, as the target user group is an older generation aged >50 years, HMDs are inconvenient to use frequently because of the weight on the head compared with a tablet.

AR brings with it 2 strengths for use in the domain of smart health care. First, AR helps engage and motivate users to use a system continuously. Once the users become familiar with using AR, they will accept the technology. Although several researchers have conducted user evaluations of AR for older adults in health care domain [12,26] and games [71,72], there is limited research on the user experience aspect of AR with older adults in the smart health care domain. Second, AR enhances the intuitiveness of data presentation [15]. As the target user group of our study was aged >50 years, the data readability on a screen is important from a user perspective. The purpose of a health-monitoring app is to convey information correctly in an easy-to-understand manner; hence, low readability would cause inconvenience for using the app. On the basis of these strengths of AR, the face filter could be helpful for older adults in a health care scenario. The face filter is a well-known technique that combines AR and facial feature detection to overlay AR contents onto the user’s face on social networking services such as Facebook, Instagram, and Snapchat. Javornik et al [73] found that using a face filter for communication can boost social interaction between people. In addition, sending older adults’ faces to their formal or informal caregivers is equal to sending complementary data to others, thereby making older adults more actively participate in their health care [74]. Despite the verified beneficial aspects of face filters, Javornik et al [73] drew their results from a younger generation aged between 19 and 35 years; hence, user evaluations of face filters with older adults are missing. Therefore, we chose to conduct a user evaluation and examine the level of acceptance of the face filter by older adults. For displaying the sensor data, Hadj Sassi and Chaari Fourati [15] had to prepare a printed marker to display AR. However, in our work, we overcame this limitation by using AR as a face filter.

### Conclusions

The need for caring services grows year by year while the resources to support them are limited. To lighten the burden on caregivers, we designed an assistive app for older adult well-being. The app supports all 3 important roles: older adults, formal caregivers, and informal caregivers. We conducted user evaluations regarding an overall impression of the app and user acceptance in terms of ease of use and usefulness of the UIs. We designed the app’s UIs using commercial apps and feedback from the participants in the iVO project. Each UI was designed to deliver data intuitively, thus enabling the user to obtain information quickly and easily. In addition, the AR is applied as a face filter to present information in a more engaging format for older adults and caregivers. Our app received a positive overall response from the user evaluation, and we identified that specific user groups preferred each UI and modality for several reasons. Accordingly, we conclude that supporting multiple UIs and interaction modalities is essential. We expect that our results will provide insight to researchers and developers on how to design an app and UI to provide a better user experience in the older adult health care domain. As future work, we intend to conduct long-term user evaluations of our app to build on end-user perspectives with specific task-based analysis.

## Appendix

Multimedia Appendix 1 Flowchart of each user interface.

Multimedia Appendix 2 User flow of each user interface.

Multimedia Appendix 3 Shared and unique features of the user interfaces.

Multimedia Appendix 4 Interview questions.

Multimedia Appendix 5 Q-Q plots of the mean of each scale per person.

Multimedia Appendix 6 Means, SDs, and CIs of the User Experience Questionnaire items.

Multimedia Appendix 7 Summary of interview answers.

## Abbreviations

AR: augmented reality
HMD: head-mounted display
IoT: Internet of Things
iVO: Internet of Things within health and care
UEQ: User Experience Questionnaire
UI: user interface
