# Supplementary figures and images for: Acceptability of a Health Care App With 3 User Interfaces for Older Adults and Their Caregivers: Design and Evaluation Study
Source: JMIR Hum Factors. 2023 Mar 8;10:e42145. doi: 10.2196/42145 (PMC10034616; doi:10.2196/42145)

# **Multimedia Appendix 1. Flowchart of each user interface.**

# Tile view


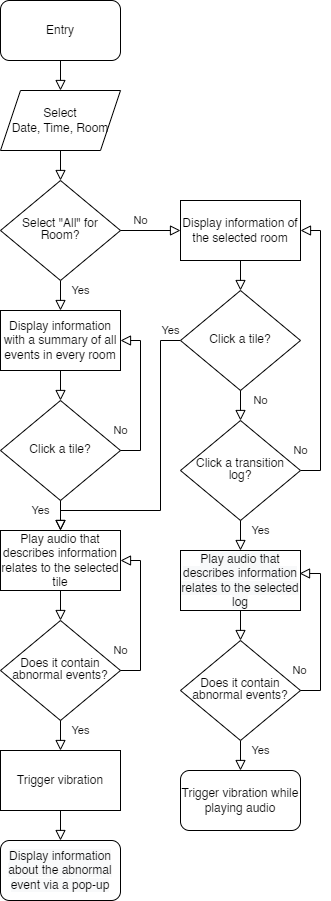


# Map view


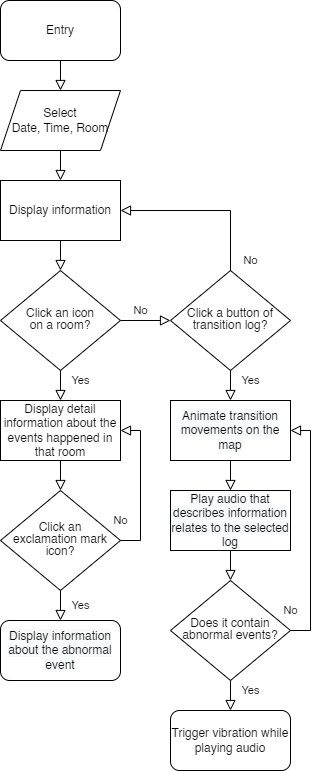


# AR view


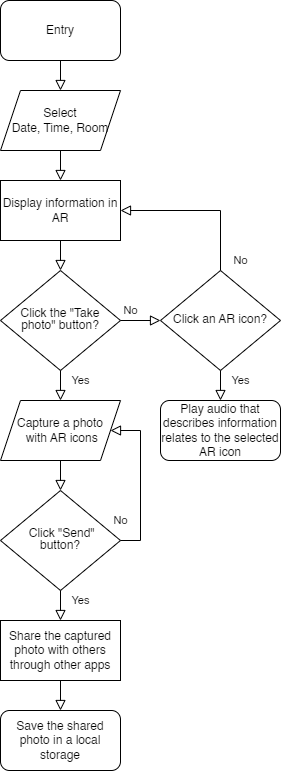

Supplement: Multimedia Appendix 1 [file humanfactors_v10i1e42145_app1.docx]

# **Multimedia Appendix 2. User flow of each user interface.**


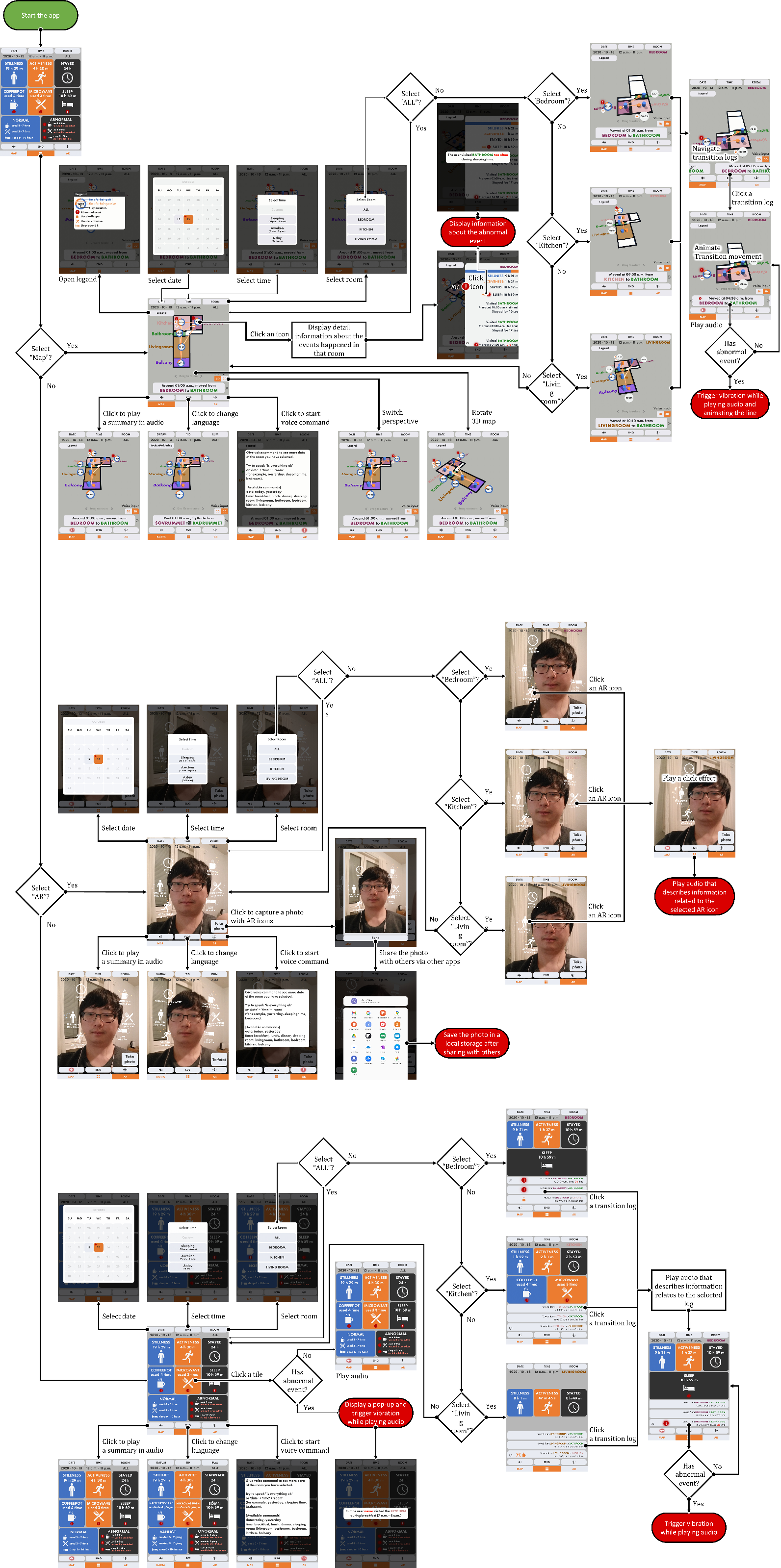

Supplement: Multimedia Appendix 2 [file humanfactors_v10i1e42145_app2.docx]
