# Supplementary material for: Acceptability of a Health Care App With 3 User Interfaces for Older Adults and Their Caregivers: Design and Evaluation Study
Source: JMIR Hum Factors. 2023 Mar 8;10:e42145. doi: 10.2196/42145 (PMC10034616; doi:10.2196/42145)
Supplement: Multimedia Appendix 3 [file humanfactors_v10i1e42145_app3.docx]

Multimedia Appendix 3. Shared and unique features of the user interfaces.

1. Target user and interaction modalities of the user interfaces.

| User interface | Target user | Input modality | Output modality |
| --- | --- | --- | --- |
|  |  |  |  |
| **Map view** | Caregivers | Touch (press and swipe)  Voice (voice command) | Visual elements  Audio  Vibration |
| **Tile view** | Relatives | Touch (press and swipe)  Voice (voice command) | Visual elements  Audio  Vibration |
| **AR view** | Older adults | Touch (press and swipe)  Voice (voice command)  Vision (facial image) | Visual elements  Audio  Vibration |

2. Unique features of each user interface.

| User Interface | Visual moving path | Transition history | Time spent in each room | Checking events | Photo capture |
| --- | --- | --- | --- | --- | --- |
|  |  |  |  |  |  |
| **Map view** | X | X | X | X |  |
| **Tile view** |  | X | X | X |  |
| **AR view** |  |  | X | X | X |
