# Supplementary material for: Acceptability of a Health Care App With 3 User Interfaces for Older Adults and Their Caregivers: Design and Evaluation Study
Source: JMIR Hum Factors. 2023 Mar 8;10:e42145. doi: 10.2196/42145 (PMC10034616; doi:10.2196/42145)
Supplement: Multimedia Appendix 4 [file humanfactors_v10i1e42145_app4.docx]

## **Multimedia Appendix 4. Interview questions**.

## Main question 1 (about overall experience)

(ENG) Were you able to easily find information that you want to see?

**(SWE)** Kunde du hitta information som du ville se?

### Sub question 1-1

(ENG) How was your experience with the application?

**(SWE)** Vad var din erfarenhet av produkten?

- Was it easy to navigate? / Var det enkelt att hitta i den?
- Was it easy to control? / Var den lätt att styra?
- What about the quality of information? Hur är det med kvaliteten på informationen?
- Was the information easy to understand? Var det lätt att förstå informationen?
- Would you like to use the application again? Skulle du vilja använda en sådan?

### Sub question 1-2

(ENG) What was easy to understand while using the application?

**(SWE)** Vad var lätt att förstå för att använda produkten?

- To find positive impressions that affected usability

### Sub question 1-3

(ENG) What was difficult / hard / unclear to understand while using the application?

**(SWE)** Vad var svårt/oklart att förstå för att använda applikationen?

- To find negative impressions that affected usability

## Main question 2 (about UI)

(ENG) Which UI did you prefer to use?

**(SWE)** Vilket användargränssnitt föredrar du att använda?

- Map UI?
- Tile UI?
- AR UI?

### Sub question 2-1

(ENG) What information were you looking for?

**(SWE)** Vilken information letade du efter?

- To identify the purpose of using a specific UI
- Overview the data? Or find detail of data?

### Sub question 2-2

(ENG) What did you like about the particular UI?

**(SWE)** Vad gillade du med användargränssnittet?

- To identify the reason for preferring a specific UI over other UI

### Sub question 2-3

(ENG) How was the chosen UI different from other UIs?

**(SWE)** Hur skilde det sig från andra användargränssnitt?

- To identify the reason for what makes the user prefer one UI over others

## Main question 3 (about interaction modality)

(ENG) Which interaction modality do you prefer to use?

**(SWE)** Vilket sätt att interagera med produkten föredrar du att använda?

- Touch input - Trycka på skärmen
- Voice input - Röststyrning
- Audio output - Ljud från produkten
- Visual output (e.g., texts, images, AR) - Visuellt t.ex. Text, bilder, AR
- Vibration

### Sub question 3-1

(ENG) (if they picked one) Was there other interaction modality you like?

**(SWE)** (om de valde en) Fanns det någon annan interaktionsmodalitet du gillar?

- To identify whether they like more than one interaction modality

### Sub question 3-2

(ENG) Can you elaborate why you liked the interaction modality that you choose?

**(SWE)** Kan du utveckla varför du gillade den interaktionsmodalitet du väljer?

- To identify the reason for preferring specific interaction modality

### Sub question 3-3

(ENG) Can you elaborate why you prefer the chosen one over other interaction modalities?

**(SWE)** Kan du utveckla varför du ogillade de andra interaktionssätten?

- To identify the reason what are the problems with other interaction modalities

### Sub question 3-4

(ENG) What is your impression about the ‘capture photo’ button?
