# Supplementary material for: Acceptability of a Health Care App With 3 User Interfaces for Older Adults and Their Caregivers: Design and Evaluation Study
Source: JMIR Hum Factors. 2023 Mar 8;10:e42145. doi: 10.2196/42145 (PMC10034616; doi:10.2196/42145)
Supplement: Multimedia Appendix 5 [file humanfactors_v10i1e42145_app5.docx]

# Multimedia Appendix 5. Q-Q plots of the mean of each scale per person.
