# Supplementary material for: Acceptability of a Health Care App With 3 User Interfaces for Older Adults and Their Caregivers: Design and Evaluation Study
Source: JMIR Hum Factors. 2023 Mar 8;10:e42145. doi: 10.2196/42145 (PMC10034616; doi:10.2196/42145)
Supplement: Multimedia Appendix 6 [file humanfactors_v10i1e42145_app6.docx]

Multimedia Appendix 6. Means, SDs, and CIs of the User Experience Questionnaire items.

Means, standard deviations (SD), and confidence intervals (CI) at 95% confidence level of 20 items in two quality aspects (QA): Pragmatic and hedonic. The questionnaire answers are transformed from the 7-stage scale to range from -3 (ie, negative words) to 3 (ie, positive words). The items with italic text have relatively high SD and low mean.

| QA | Scale | Item | | Mean (SD) | 95% CI |
| --- | --- | --- | --- | --- | --- |
|  |  | Negative word | Positive word |  |  |
|  |  |  |  |  |  |
| **Pragmatic** | |  |  |  |  |
|  | Efficiency | Slow | Fast | 1.76 (1.39) | 1.19-2.33 |
|  |  | Inefficient | Efficient | 2.04 (0.98) | 1.64-2.44 |
|  |  | Impractical | Practical | 1.92 (1.29) | 1.39-2.45 |
|  |  | *Cluttered* | *Organized* | 1.60 (1.76) | 0.88-2.32 |
|  | Perspicuity | Not understandable | Understandable | 2.08 (1.32) | 1.53-2.63 |
|  |  | Difficulty to learn | Easy to learn | 1.88 (1.42) | 1.29-2.47 |
|  |  | Complicated | Easy | 1.72 (1.28) | 1.19-2.25 |
|  |  | *Confusing* | *Clear* | 1.84 (1.82) | 1.09-2.59 |
|  | Dependability | *Unpredictable* | *Predictable* | 0.60 (1.26) | 0.08-1.12 |
|  |  | Obstructive | Supportive | 2.60 (0.65) | 2.33-2.87 |
|  |  | Not secure | Secure | 1.60 (1.19) | 1.11-2.09 |
|  |  | Does not meet expectations | Meet expectations | 2.16 (0.99) | 1.75-2.57 |
| **Hedonic** | |  |  |  |  |
|  | Stimulation | Inferior | Valuable | 2.20 (1.15) | 1.72-2.68 |
|  |  | Boring | Exciting | 2.04 (0.98) | 1.64-2.44 |
|  |  | Not interesting | Interesting | 2.32 (0.80) | 1.99-2.65 |
|  |  | Demotivating | Motivating | 2.16 (0.80) | 1.83-2.49 |
|  | Novelty | Dull | Creative | 1.84 (1.18) | 1.35-2.33 |
|  |  | Conventional | Inventive | 2.28 (0.84) | 1.93-2.63 |
|  |  | Usual | Leading edge | 2.32 (0.95) | 1.93-2.71 |
|  |  | Conservative | Innovative | 2.20 (1.26) | 1.68-2.72 |
