# Supplementary material for: Acceptability of a Health Care App With 3 User Interfaces for Older Adults and Their Caregivers: Design and Evaluation Study
Source: JMIR Hum Factors. 2023 Mar 8;10:e42145. doi: 10.2196/42145 (PMC10034616; doi:10.2196/42145)
Supplement: Multimedia Appendix 7 [file humanfactors_v10i1e42145_app7.docx]

# Multimedia Appendix 7. Summary of interview answers.

# General Impression

## Informative data

| *Even a small event like visiting a toilet can be checked that my parent may not remember anymore* [P1]  *If I visualize myself using this tool, I will probably use it when I am walking between older adults. Because when I am there (ie, older adult's residence), I want to focus on the person in front of me. Maybe I will go deep if somebody tells me, “I slept badly” or “I am very hungry.” Then, I would check if there were any abnormal activities there, which I don't remember that I had checked before. Otherwise, I will probably check it before going there so I can prepare something if it is there* [P5].  *It seems like great information from the beginning, and there is more comprehensive information in any case* [P8].  *I can feel safer if I have this. Someone knows that I am still moving around. For instance, my children can see that I am moving. If you develop dementia, perhaps, you don't know if you've eaten or not. This can tell if you did it* [P13].  *I think it's very innovative, and I think it's gonna be very useful because we don't have time to visit our older parents* [P18].  *When home care service comes home of people, they want to check information. So it is also something that they could use this to have some kind of logs for them to use it for [knowing] what is happened* [P19]. |
| --- |

## Intuitive UI design

| *I think it is easy enough [to find information]. It mostly seems straightforward. I liked the tile view for easy access to all information. It gives me everything what I need to know straight up* [P5].  *It looks like a good way to visualize the information. The information presented in each [room] is relevant. It is more intuitive than abstract* [P10].  *It feels intuitive and easy to understand. The map view is where you can see the apartment, and you can see exactly more precisely* [P11].  *[Because of the colors] It’s very easy to understand what it is what* [P14]. |
| --- |

## Ease of use

| *I think it is easy to find [information what I am looking]* [P3].  *Learning is quite easy. Even if I don't get it all initially, I quickly understand it* [P4].  *I think I will be able to use it without anyone telling me how to use it* [P26]. |
| --- |

## Age dependency

| *It is very difficult to use for older adults who are not familiar with this kind of system* [P4].  *Maybe the next generation can get into use it, but not for this [older] generation yet. Not only hard to motivate them to use this kind of technology, but they also do not have knowledge of this, even if they have motivation. They can forget [due to like dementia] how to use it, and what remains is the knowledge they used quite often for a long time, like a memory that lives in their backbone, and this kind of technology is not the one that they have been used* [P5].  *[This app is] suitable depending on the user. …Not only the age but also the skills that the user has affected the experience. The younger generation can enthusiastically use it* [P7]*.* |
| --- |

## Lack of design clarity

### Ambiguity of data visualization in the map view

| *In map view, the bars on icons don't represent exact time windows of behavior in first look* [P6].  *In map view, the proportion of a bar on the icons is vague to understand the exact time duration* [P6]. |
| --- |

### Vague motivation for utilization

| *Since audio output are same as the text information, I wouldn't use audio output* [P6].  *I don’t get a reason why this function (ie, rotating the map) exists* [P10].  *I don't really understand why my mother or father would use this [tile] view. I don't see why they want to check how often they used the microwave or coffeepot or something. …I can imagine that if a person has a medicine, and if someone has taken medicine. Some kind of app that you can check for it [would be needed]. Because I know it is something both her (ie, P19’s wife's) parent and my father have. It could be a pillbox, which is very important in their life* [P19]. |
| --- |

### Lack of consideration for user experience in UI design

| *The speech speed is little bit fast. It would be better to bit slower* [P1].  *One thing would be “too many things to click”* [P3].  *In the tile view, it says how much [time spent]. But in the map view, it doesn't unless a user clicks the icon* [P6].  *The available voice commands are not intuitively presented* [P6].  *It would be better if the application can accept natural language as a voice command* [P9]. |
| --- |

# User Acceptance

## UI preferences

### Map view

#### Older adults

| *The map view gives a feeling that I am in the room, whereas the tile view is more abstract* [P10].  *You can see the movements around all the time* [P15].  *I think this (ie, map view) is more interesting because you can also see the movements. …It's very easy to see movements* [P16].  *So, the map, together with time [in a text] and room [in the 3D map] and this lot of information you get [from the detail view], are quite easy to understand. Not so difficult to understand. Not at all* [P16].  *It has both graphical information (ie, icons, lines, and cylinders) and text that I can read [for more details]* [P16].  *This gives more information. I can also see the movements between rooms* [P25]. |
| --- |

#### Formal caregiver

| *The map view is probably more useful in a meeting that is placed every 3 - 6 months with both patients (ie, older adults) and clients (ie, relatives) to show what happened* [P5]. |
| --- |

#### Informal caregivers

| *Because it has more visual things that I can quickly read from the maps with icons and texts* [P3].  *I want to check 3 - 4 times per day, so not much of details are needed. I want to see the level of activity daily without looking at details* [P7].  *Cylinders on the map helped to understand the information quickly* [P7].  *Instead of clicking each tile, it gives overview* [P9]. |
| --- |

### Tile view

#### Older adults

| *The tile view is easy to understand* [P2].  *You can get an overview, but you can also get a detail* [P13].  *It gives a very good overview* [P14]. |
| --- |

#### Formal caregiver

| *Caregivers don't need to know that much of details per day but in an overview of a longer period* [P5].  *Especially the tile view plainly gives me all the information on one page* [P5]. |
| --- |

#### Informal caregivers

| *The map view presents less detailed information in the first view. …In tile view, it says how many, but in map view, it doesn't unless a user clicks the icon* [P6].  *If you have just a few minutes to check, you can just see bullet points (ie, each tile). …If you have a look at the map, you have to think a little bit [to understand]* [P18].  *A fast overview of what is happening. I mean, I can imagine if I had this tablet at home and then could walk by. You see something is happening* [P19].  *I prefer the tile view because I am used to read Excel files and columns* [P23]. |
| --- |

### AR view

#### Older adults

| *If this has an interactive communication system, like a face-call, it would be really great* [P10].  *If I have some problem (ie, broke something on my body) I can share my status with my daughter quickly* [P10].  *If this feature can be used with Skype, like a phone call with face, it would be better* [P11].  *I think it is very good that it has the possibility of interacting with others* [P14]. |
| --- |

#### Formal caregiver

| *It is useful for the caregiver and relative perspective. Because even if we can still access older adults' information from other UIs, photo with their face presents us that they are active, and it also helps the person feel that they are more involved in healthcare* [P5]. |
| --- |

#### Informal caregivers

| *AR is not for a relative, but I would like to ask my parent to send his photo at specific times to check whether he is fine. For example, each mealtime in the day. It is also good for older adults. That feature (ie, capturing and sharing a facial image) enables interaction between older adults, caregivers, and relatives like a social interaction* [P7].  *The AR view is mainly good for older adults to show what is collected. …It is very good to let them be involved in this [healthcare] by themselves* [P9].  *I think it was good. You can see what the facial expression says. …You can get more information from the picture than what you actually do [from the texts]* [P17]*.*  *I think it’s good if you are alone and an older person. Maybe you have to express yourself. Then, you just contact your children and grandchildren, and if they say, “What did you do today, father.” Then, you can just put this picture and just say, “I don't know what I did.” This picture tells them, and they can see how you really look there. Because you can send this picture, and they see your activity. And then in the communication, [they will say like,] “Wow, I saw you are more active today than last day”* [P18]. |
| --- |

## Interaction modality preferences

### Input modality

#### Touch

##### Older adults

| *Clicking with a finger is most familiar because I did it on my phone and my iPad* [P14]. |
| --- |

##### Informal caregivers

| *In map view, I prefer to use touch (Because) the map view requires touch to navigate many icons* [P7].  *Touch is simple to use. I don't need to learn how to [use] compared to voice command* [P9].  *It’s more like how we are used to work with apps or stuffs* [P19]. |
| --- |

#### Voice

##### Older adults

| *Graphical user interface needs to be intuitivel; otherwise, we would stop using it* [P10]. |
| --- |

##### Informal caregivers

| *Maybe voice command can be used for someone who don’t know how to interact with the UI* [P6].  *If you are blind, I can imagine you have a different perspective [on the value of voice interaction] than I do* [P19]. |
| --- |

#### Touch and Voice

##### Older adults

| *Because it is easy to press* [P2].  *Right now, the finger is best for me. But perhaps in 20 years, I will go something else. So it's very good that it has all the possibilities [for interaction]* [P13]. |
| --- |

##### Informal caregivers

| *Maybe I would like to use every interaction modality first, and once I get used to use some of them, I may stick with some of them only* [P3]. |
| --- |

### Output modality

#### Visual element

##### Older adults

| *The map view gives a feeling that I am in the room, whereas the tile view is more abstract* [P10].  *It feels intuitive and easy to understand. The map view is where you can see the apartment, and you can see exactly more precisely* [P11].  *[Because of the colors] It’s very easy to understand what it is what* [P14].  *You can see the movements around all the time* [P15].  *I think this (ie, map view) is more interesting because you can also see the movements. …It's very easy to see movements* [P16].  *It has both graphical information (ie, icons, lines, and cylinders) and text that I can read [for more details]* [P16]. |
| --- |

##### Formal caregiver

| *Personally, I probably do not use anything except reading the page (ie, the tile view). But, I can see other people more like to see graphical things [in the map view] to understand more easily. …In time-wise, reading [texts] is much faster rather than listening to the audio output* [P5]. |
| --- |

##### Informal caregivers

| *Because it has more visual things that I can quickly read from the maps with icons and texts* [P3].  *Cylinders on the map helped to understand the information quickly* [P7].  *I think it’s (ie, the tile view) very overviewing, and you just [click a tile] and see [the tiles] one by one quickly* [P18].  *It’s more like how we are used to work with apps or stuffs* [P19].  *I prefer the tile view because I am used to read Excel files and columns* [P23]. |
| --- |

#### Audio

##### Older adults

| *I don't feel comfortable with computer … Touching it will probably be harder* [P12]. |
| --- |

##### Formal caregiver

| *Maybe just reading the same information is useful to older adults themselves or people who do not like to read the text* [P5]. |
| --- |

##### Informal caregiver

| *But if the audio output provides more information than the text, it would be beneficial even if that information can also be presented in graphics* [P6]. |
| --- |

#### Visual and Audio

##### Older adults

| *Right now, I prefer to read it. But later, it might be better to hear the voice* [P13].  *It depends on us if we start to develop problems with my eyes or with hearing. I will prefer one or the other* [P24]. |
| --- |
